# MERS-CoV virus-like particles produced in insect cells induce specific humoural and cellular imminity in rhesus macaques

**DOI:** 10.18632/oncotarget.8475

**Published:** 2016-03-30

**Authors:** Chong Wang, Xuexing Zheng, Weiwei Gai, Yongkun Zhao, Hualei Wang, Haijun Wang, Na Feng, Hang Chi, Boning Qiu, Nan Li, Tiecheng Wang, Yuwei Gao, Songtao Yang, Xianzhu Xia

**Affiliations:** ^1^ College of Wildlife Resources, Northeast Forestry University, Harbin, China; ^2^ Key Laboratory of Jilin Province for Zoonosis Prevention and Control, Institute of Military Veterinary, Academy of Military Medical Sciences, Changchun, China; ^3^ School of Public Health, Shandong University, Jinan, China; ^4^ Jiangsu Co-innovation Center for Prevention and Control of Important Animal Infectious Diseases and Zoonoses, Yangzhou, China

**Keywords:** Middle East respiratory syndrome coronavirus, vaccine, virus-like particles, nonhuman primates, immune response

## Abstract

Middle East respiratory syndrome coronavirus (MERS-CoV) causes severe respiratory disease in humans with a case fatality rate of over 39%, and poses a considerable threat to public health. A lack of approved vaccine or drugs currently constitutes a roadblock in controlling disease outbreak and spread. In this study, we generated MERS-CoV VLPs using the baculovirus expression system. Electron microscopy and immunoelectron microscopy results demonstrate that MERS-CoV VLPs are structurally similar to the native virus. Rhesus macaques inoculated with MERS-CoV VLPs and Alum adjuvant induced virus-neutralizing antibodies titers up to 1:40 and induced specific IgG antibodies against the receptor binding domain (RBD), with endpoint titers reaching 1:1,280. MERS-CoV VLPs also elicited T-helper 1 cell (Th1)-mediated immunity, as measured by ELISpot. These data demonstrate that MERS-CoV VLPs have excellent immunogenicity in rhesus macaques, and represent a promising vaccine candidate.

## INTRODUCTION

Middle East respiratory syndrome coronavirus (MERS-CoV) is a betacoronavirus infecting humans, but MERS-CoV disease is markedly different from other human coronaviruses including HCoV-HKU1, HCoV-NL63, HCoV-OC43 and HCoV-229E, which are known to cause mild respiratory infections. Rather, MERS-CoV infection is similar to Severe Acute Respiratory Syndrome coronavirus (SARS-CoV), as disease is associated with acute respiratory distress syndrome. In addition, MERS-CoV infection can result in kidney failure, ultimately leading to death [1–3]. As of 29 October 2015, 628 deaths from 1635 cases of MERS-CoV had been identified worldwide, a case fatality rate of 39.02% [4]. Human infections with MERS-CoV typically occur in countries located in the Middle East, but in May 2015 an outbreak was reported in South Korea, in which three super-spreaders were found to be responsible for the majority of infections, as well as evidence of tertiary human-to-human transmission [5]. As such, there is an urgent need for the development of an efficacious vaccine against MERS-CoV.

Past vaccine strategies against viral pathogens comprise of live-attenuated viruses that were passaged in animal hosts or cell lines before immunization, however these vaccines are not always sufficiently immunogenic, and there are safety concerns associated with the use of live-attenuated vaccines in some populations, particularly the young, old, pregnant or immunocompromised. Inactivated vaccines are safe for use, but typically only induce humoural immunity and very low levels of cell-mediated responses. There are no reports of inactivated MERS-CoV being tested as a vaccine, but an inactivated SARS-CoV vaccine appears to have little effect in mice and domestic ferrets [6–8]. Recently, candidate vaccines are genetically engineered to be replication-deficient or avirulent before use, therefore eliminating the concern that live-attenuated vaccines could revert to virulence. A recombinant MERS-CoV lacking the E structural protein was previously developed as a candidate vaccine; however, its protective efficacy has not yet been demonstrated [9].

Other MERS-CoV candidate vaccines currently being developed include spike (S) protein nanoparticles [10], modified vaccinia virus vectors [11] and immunogens based on the full-length S DNA and subunit protein S1 [12], all of which have been shown to be able to induce neutralizing antibodies against MERS-CoV. Virus-like particles (VLPs) are protein-only subunit vaccines that emulate the morphology of the native virus. Compared with inactivated or live-attenuated virus vaccines, VLPs are able to induce robust humoural and cellular immune responses without the risk of reversion to virulence [13, 14]. Furthermore, VLPs for any pathogen can be generated under BSL-2 conditions.

In this study, we constructed recombinant baculovirus co-expressing the S, envelope (E) and membrane (M) genes. Infection of Sf9 cells with this recombinant baculovirus resulted in the successful assembly of MERS-CoV VLPs. We then confirmed the structural integrity of VLPs and evaluated the immunogenicity of MERS-CoV VLPs as a vaccine candidate in rhesus macaques.

## RESULTS

### Generation of recombinant baculovirus and MERS-CoV VLPs

**Figure 1:**
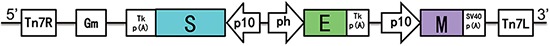
Schematic of the recombinant baculovirus expressing MERS-CoV S, E and M genes The Tn7 regions, gentamicin resistance gene (Gm), HSV tk polyadenilation signal [Tk p (A)], p10 promoter (p10), polyhedrin promoter (ph), SV40 polyadenylation signal [SV40 p (A)], and MERS-CoV isolate Al-Hasa_15_2013 genes are shown. S, spike protein; E, envelope protein; M, membrane protein.

The MERS-CoV S, E, M genes were cloned into the modified pFastBacDual vector in the locations shown (Figure 1) and the recombinant plasmid was confirmed by enzyme digestion analysis as well as DNA sequencing and the recombinant plasmid was tranfected into Sf9 cells to obtain recombinant baculovirus. The titer of recombinant baculovirus stocks at the third passage was determined to be 3.7×10^7^ infectious units (IFU)/ml. Infection of Sf9 cells with recombinant baculovirus yielded MERS-CoV VLPs, which were purified with a discontinuous sucrose gradient for further studies.

### Production and identification of MERS-CoV VLPs produced in insect cells

**Figure 2:**
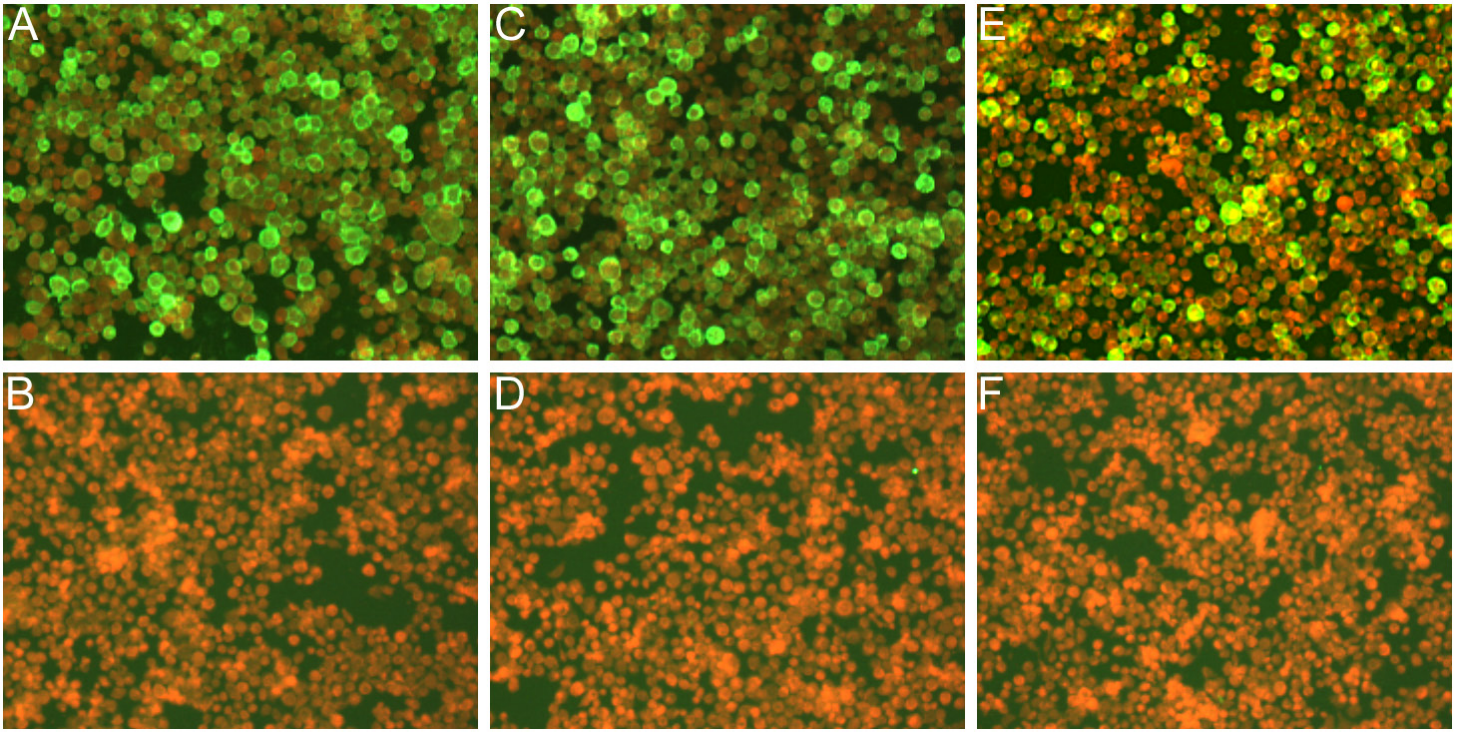
IFA detection of expression of the triple baculoviruses in Sf9 infected cells Cells were infected with the recombinant baculoviruses in **A, C, E**. and were mock infected in **B, D, F**. Cells were detected with anti-S polyclonal sera in A and B, anti-M polyclonal sera in C and D, and anti-E polyclonal sera in E and F, respectively.

**Figure 3:**
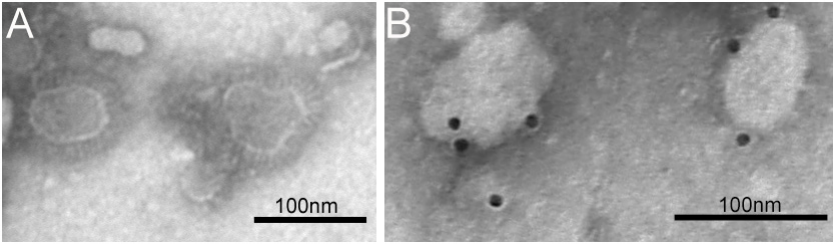
TEM and IEM analysis of MERS-CoV VLPs **A.** MERS-CoV VLPs produced by infected Sf9 cells and purified by sucrose gradientwere stained with 1% sodium phosphotungstate. Bar = 100 nm. **B.** The VLPs were incubated with anti-S polyclonal antibody and probed using a gold-labeled goat anti-mouse IgG antibody.

**Figure 4:**
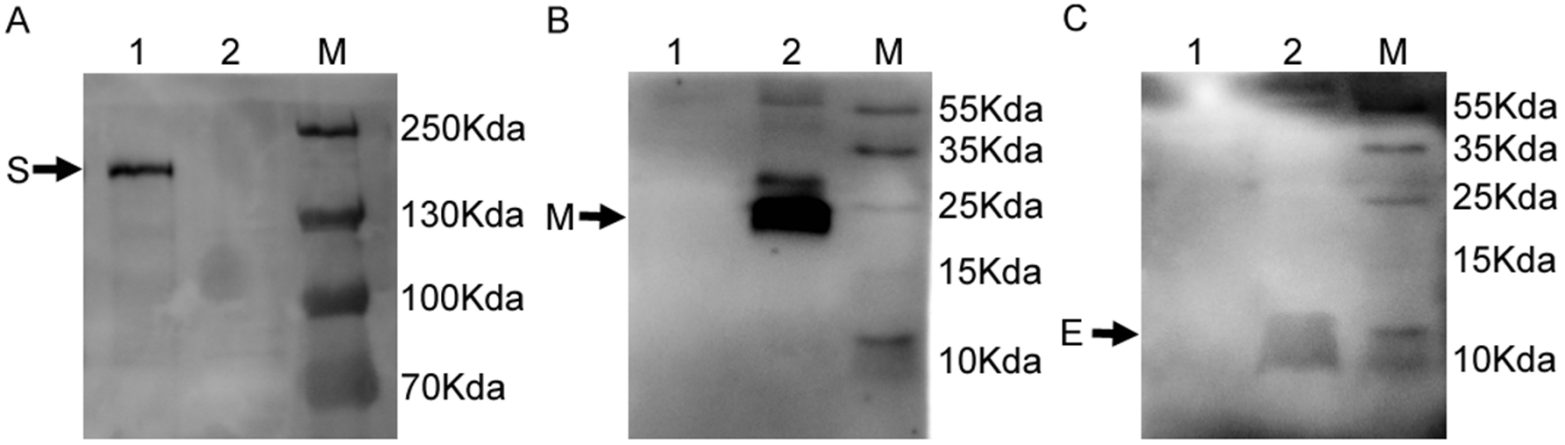
Detection of expression of S, E and M proteins in MERS-CoV VLPs MERS-CoV VLPs analyzed by WB using **A.** rabbit anti-S polyclonal antibody (lane 1 MERS-CoV VLPs; lane 2 lysate from pFastBacDual infected cells served as a control) and **B.** mouse anti-M polyclonal antibody (lane 1 lysate from pFastBacDual infected cells served as a control; lane 2 MERS-CoV VLPs) and **C.** mouse anti-E polyclonal antibody (lane 1 lysate from pFastBacDual infected cells served as control; lane 2 MERS-CoV VLPs).

Immunofluorescence assay confirmed the recombinant baculovirus was expressed in Sf9 cells. The results of immunofluorescence studies demonstrated the expression of three structural proteins (Figure 2). The morphology of MERS-CoV VLPs was investigated by electron microscopy. Under TEM, the diameters of MERS-CoV VLPs were approximately 100 nm, and spikes were readily observable around the spherical particles (Figure 3A). To ensure that S was incorporated into the VLPs, immunoelectron microscopy was performed with a gold-tagged antibody against the RBD of S. Results show that the gold particles appear on the MERS-CoV VLPs (Figure 3B), demonstrating that the particle contain S. Each composition of the MERS-CoV VLPs was confirmed by Western blot, and bands corresponding to S, M and E in MERS-CoV VLPs were observed (Figure 4). These results demonstrate that MERS-CoV VLPs autonomously assemble in insect cells that have been infected by our recombinant baculovirus, and are structurally similar to the native virus [15].

**Figure 5:**
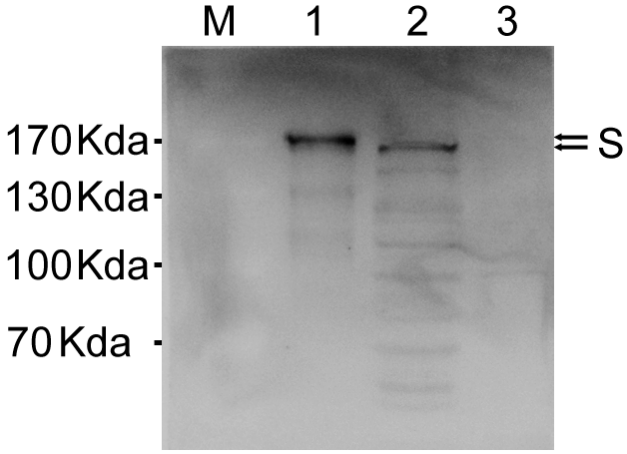
Comparison of spike glycoprotein expression in insect and mammalian cells Western blot analysis of MERS-CoV VLPs (lane 1) and cell lysate from pcDNA3.1-MERS-S transfected BSR cells at 72 h after transfection (lane 2), lysate from uninfected BSR cells served as controls (lane 3). S protein was analyzed by immunoblotting using a polyclonal anti-S antibody.

Our next objective was to answer whether expression from insect or mammalian cells have any difference on glycosylation levels on the S protein. To investigate this, the pcDNA3.1-MERS-S transfected BSR cell lysates were compared with purified MERS-CoV VLPs by Western blot. S expressed in insect cells was slightly larger than those produced in mammalian cells (Figure 5).

### Antibody responses in vaccinated rhesus macaques

**Figure 6:**
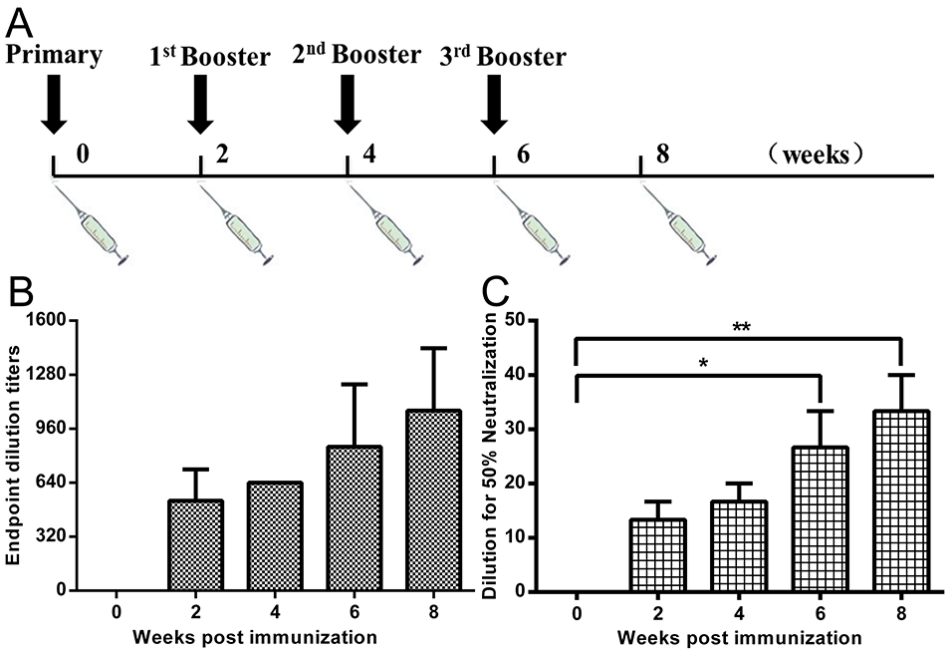
NHP immunization procedure, RBD-specific antibody and neutralizing antibodies against MERS-CoV infection **A.** Rhesus macaques (n=3) were vaccinated IM with 250 μg of MERS-CoV VLPs and Alum adjuvant (at 0, 2, 4, 6 weeks) and one group was treated with equivalent volumes of PBS as a control (n=3). Blood samples were collected from the femoral vein of monkeys before immunization and two weeks after each immunization. **B.** ELISA results show that immunized monkeys were able to induce a robust humoral response specific to RBD, with serum IgG titers of up to 1:1,280 after the fourth immunization. **C.** Rhesus macaque blood samples were collected from the femoral vein of monkeys two weeks after last immunization. MERS-CoV live virus-based inhibition assay were taken in Vero E6 cells in triplicate. The titers were determined as the highest serum dilutions that completely prevent CPE in at least 50% of the wells (NT_50_) and are expressed as mean +/- SEM. Statistical analysis between the two groups were analyzed by t-test, in which a p-value of less than 0.05 was considered statistically significant (** p<0.05).

To evaluate the humoral immune response in immunized nonhuman primates (NHPs), we measured serum neutralizing antibody against MERS-CoV live virus. The titers were determined as the highest serum dilutions that completely prevent CPE in at least 50% of the wells, and the virus-neutralizing antibodies titers up to 1:40 after fourth immunization (Figure 6A and 6C). We also measured serum IgG antibody concentrations against the RBD protein using ELISA. Results showed that NHPs immunized with the VLPs and Alum adjuvant were able to induce considerable amounts of IgG antibody, with endpoint titers up to 1:1,280 when measured at 8 weeks after vaccination (Figure 6B). In contrast, MERS-CoV virus-neutralizing antibodies or RBD-specific IgG was not detected in mock-vaccinated NHPs.

### Cell-mediated responses in NHPs

**Figure 7:**
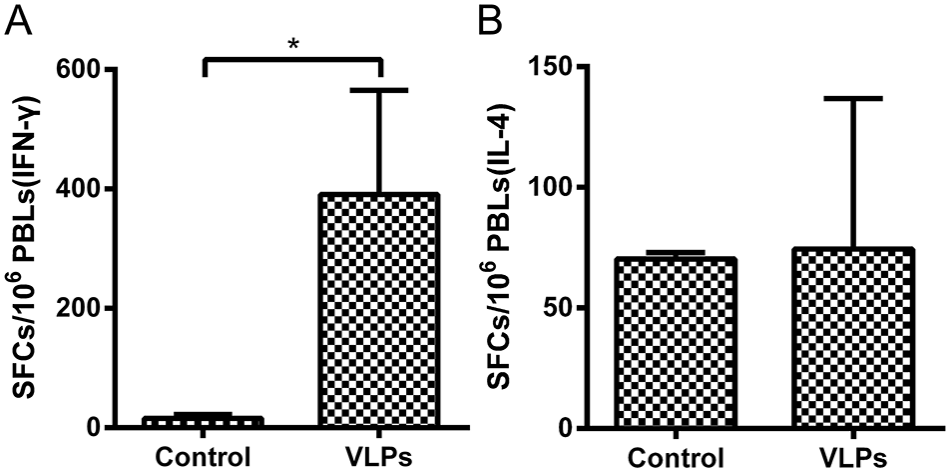
Enzyme-linked immunospot assays of IFN-γ and IL-4 secretion in NHPs PBMCs were isolated from NHPs and stimulated with the purified RBD of the MERS-CoV S protein. PBMCs secreting **A.** IFN-γ or **B.** IL-4 were quantitated using ELISpot assay. The data represent the mean +/- standard deviation (SD) of SFCs per million. Statistical analysis between the two groups were analyzed by t-test, in which a p-value of less than 0.05 was considered statistically significant (* p< 0.05).

To assess T-cell responses in NHPs following vaccination, the extent of IFN-γ and IL-4 secretion from the PBMCs of NHPs were determined using ELISpot assays. Results show that the PBMCs of VLP-vaccinated NHPs had significantly higher release of IFN-γ upon stimulation with purified RBD antigen, compared to PBMCs from mock-vaccinated animals (Figure 7A). In contrast, IL-4 secretion was not substantially different between the two groups (Figure 7B).

## DISCUSSION

VLP production via the baculovirus expression system platform in insect cells is a tried and tested strategy for vaccine development. Several VLP-based prophylactics have been successfully produced, tested, and commercialized. These include Cervarix™ (GlaxoSmithKline), which is a vaccine against human papillomavirus (HPV) and thus cervical cancer [16]; FluBlok®, which is a trivalent hemagglutinin vaccine approved by the FDA [17]. Ingelvac, CircoFLEX® and Circumvent® PCV are two approved animal vaccines against porcine circovirus (PCV) type 2 [18]. Furthermore, a variety of influenza VLPs also demonstrate excellent immunogenicity. Novavax (MD, USA) has produced a VLP-based vaccine targeting H1N1 influenza virus, which has recently shown encouraging results in a Phase II trial [19].

Past findings showed that SARS-CoV VLPs could be readily assembled by the co-infection of insect cells with baculoviruses expressing M and E. The co-expression of S, E and M by a single recombinant virus allowed for the efficient assembly and release of VLPs not only closely resembled SARS-CoV in size and particle morphology, but also virus morphogenesis [20]. However, in our study co-infection of the MERS-CoV S and M recombinant baculoviruses in insect cells did not result in VLPs formation, as assessed by TEM (data not shown). Rather, we demonstrated that VLPs consisting three structural envelope proteins (S, E, M) can be generated in insect cells with similar morphology to MERS-CoV virions. The expression of S protein from insect cells was slightly larger than that from mammalian cells, suggesting that the glycosylation of our VLPs is complete. To our knowledge, this is the first report which showed the successful generation of MERS-CoV VLPs consisting of immunogenic proteins.

We currently lack an animal model in which challenge with MERS-CoV will cause uniform lethality [21, 22]. The NHPs develop transient infection but do not die from infection, whereas transgenic mice overexpressing DPP4 die from MERS-CoV infection [23, 24]. As such, the protective efficacy of candidate vaccines against MERS-CoV cannot be evaluated in animals under the most stringent conditions. Due to the immunological similarity of rhesus macaques to humans [21, 25], the next best choice is to determine whether our MERS-CoV VLPs are able to elicit robust levels of immunogenicity in NHPs, in hope that humans will react to the vaccine in a similar manner. This rationale is supported by a past study that also used rhesus macaques for the evaluation of a candidate countermeasure against MERS-CoV infection [26].

Our results showed that MERS-CoV VLPs are immunogenic, able eliciting robust levels of specific humoural and cell-mediated immunity in vaccinated NHPs. After immunization with Alum adjuvant, rhesus macaques develop virus-neutralizing antibodies and high levels of MERS-CoV specific IgG antibodies against the RBD. Vaccination with MERS-CoV VLPs also resulted in T-cell responses to RBD, as assessed by ELIspot assay of peptide-stimulated PBMCs. IFN-γ, but not IL-4 secretion was detected from PBMCs, suggesting that VLPs elicited a Th1-mediated response. Since both specific humoral immunity and cell immunity is indispensable for effective vaccination [27], this suggests that our VLP-based vaccine has potential to be protective in humans against MERS-CoV challenge. Future experiments should involve efficacy testing in rhesus macaques, transgenic mice overexpressing DPP4, and the safety testing of this VLP-based vaccine candidate in phase I clinical trials.

## MATERIALS AND METHODS

### Construction of recombinant baculoviruses

In order to acquire a ternary promoter transfer plasmid, a gene segment contains a terminator and a p10 promoter sequence flanking with several restriction enzyme sequences was synthesized. The modified sequences were cloned into the *Bam*HI and *Hind*III sites of the pFastBacDual vector (Invitrogen, Carlsbad, CA), under the control of the polyhedron promoter. The S, E and M gene sequences of MERS-CoV isolate Al-Hasa_15_2013 were obtained from NCBI (GenBank accession No. KF600645). The genes were codon-optimized for the best possible expression levels in insect cells and biochemically synthesized (Sangon Biotech, China). The full-length gene encoding the S protein was inserted into *Xho*I and *Kpn*I sites, under the control of the p10 promoter, the E gene was cloned into the *Bam*HI and *EcoR*I sites, under the control of the polyhedron promoter, and the M gene was inserted into the *Sal*I and *Not*I sites, under the control of the additional p10 promoter, generating the recombinant plasmid pFastBacDual-SEM. The plasmid was examined by restriction enzyme analysis to confirm the correct orientation of the insertions. Bacmid transfer plasmids expressing site-specific transpositions of the S, E and M genes were then transformed into *E.coli* DH10Bac competent cells to generate recombinant bacmids. The recombinant bacmids were authenticated by PCR using pUC/M13 Forward and Reverse primers, and then transfected into Sf9 insect cells using Liposome 2000, following the Bac-to-Bac Expression Systems manual (Invitrogen, USA). Supernatant containing recombinant baculovirus was harvested 5 days after transfection as the viral stock.

### Generation of VLPs

Titers of baculovirus stocks were determined using a rapid titration kit (BacPakBaculovirus Rapid Titer Kit; Clontech, USA). Sf9 insect cells were maintained as suspension cultures in serum-free SF900II medium (Life technologies, USA) at 27°C, with agitation at a speed of 120 rpm. MERS-CoV VLPs were produced by infecting Sf9 cells with recombinant baculovirus at a multiplicity of infection (MOI) of 0.5, and harvested at 96 h post infection (hpi). Suspensions were first centrifuged to remove cell debris, and the supernatants were then ultracentrifuged at 100,000 g for 1 h to obtain the VLPs pellet, before purification by a 30–40–50% discontinuous sucrose gradient. Bands between 30-40% sucrose, which represent MERS-CoV VLPs, were collected.

### Indirect immunofluorescence assay (IFA)

Immunofluorescence assay was performed as previously described [20]. Briefly, Sf9 cells were infected with the recombinant baculovirus for 48 h, and then fixed with 4% paraformaldehyde for 20 minutes at room temperature. Subsequently, cells were incubated with the primary antibodies (mouse polyclonal antibodies against MERS-CoV S, E and M protein, respectively) that contained 1% bovine serum albumin for 1 h. After three washes with PBS containing 0.05%Tween 20 (PBST), secondary antibodies (FITC-labeled goat against mouse IgG) and 0.3% Evans blue were added for 50 minutes at room temperature. The cells were observed using a fluorescence microscope after washing.

### Electron microscopy

MERS-CoV VLPs were loaded onto grids, kept at room temperature for 5 min, stained with 1% sodium phosphotungstate, and then examined with transmission electron microscope (TEM). For immunoelectron microscopy (IEM), the MERS-CoV VLPs were loaded onto formvar-coated grids after removal of excess sample solution, incubated with mouse anti-S antibodies, labeled with gold-tagged goat anti-mouse IgG antibody (Sigma-Aldrich, SaintLouis, MO, USA), and then observed with the TEM.

### Western blot

Purified MERS-CoV VLPs were transferred onto a Polyvinylidene fluoride (PVDF) membrane (Immobilin-P, Millipore, USA) after SDS-PAGE under denaturing conditions for Western blotting with anti-S, E, M mouse polyclonal antibodies.

### Comparison of spike glycoprotein expression in insect and mammalian cells

Briefly, BSR cells were transfected with pcDNA3.1-MERS-S and cells were lysed 3 days later. The lysates from BSR cells and MERS-CoV VLPs from Sf9 cells were compared by using anti-S polyclonal sera by Western blotting.

### Immunization studies

Six rhesus macaques were randomized into two groups. One group was vaccinated intramuscularly (IM) in the gastrocnemius muscle with 250 μg of MERS-CoV VLPs and mixed with 250 μg Alum adjuvant (Thermo, USA) per animal, and the other group was given an equivalent volume of PBS as a control. Identical vaccinations were then repeated at 14-day intervals three more times for both groups. Blood samples were obtained from the femoral vein of animals prior to each vaccination.

### RBD-specific antibody measurement in the sera of rhesus macaques

RBD-specific antibodies from the sera of immunized and control monkeys were measured by indirect ELISA. Genes encoding RBD protein (spike residues 358-662) [28, 29] of MERS-CoV were amplified by PCR using synthesized MERS-CoV S sequences (GenBank accession no. KF600645) as the template and cloned into the pET-30a expression vector. The proteins were purified by Ni-NTA affinity chromatograph column (Thermo, USA), according to manufacturer instructions. Briefly, 96-well microtiter plates (Corning Costar, USA) were pre-coated with 100 μL of purified RBD antigen at a concentration of 1 μg/mL diluted in 0.05 mol/L carbonate-bicarbonate buffer (pH 9.6) and incubated at 4°C overnight. After the plates were blocked for 2 h at 37°C, 100 μL of serum samples 2-fold serially diluted were added to the wells, and incubated at 37°C for 1 h. After three washes with PBST, 100 μL of HRP-labeled goat polyclonal antibody against monkey IgG (Abcam, UK) was added (diluted 1:20,000), and incubated at 37°C for 1 h. After washing with PBST three times, 100 μL of the substrate 3, 3′, 3, 5′-tetramethylvenzidine (TMB) (Sigma, USA) was added to each well, incubated for 30 min, and then stopped with 50 μL of 2 M H_2_SO_4_. Optical density values were measured using an ELISA plate reader at a wavelength of 450 nm (Bio-Rad, USA).

### MERS-CoV inhibition assay

Neutralizing antibody titers of NHPs sera detected as previously described [30]. Briefly, the 2-fold serially diluted serum samples of NHPs were incubated with 100 TCID_50_ of virus for 1 h at 37°C before added to the monolayer of Vero E6 cells in triplicate. Virus supernatant was removed after 1 h of culture at 37°C then replaced with fresh medium. Cells were observed daily for cytopathic effect (CPE) and recorded on day 3 post-infection. The highest dilution of the monkey sera that completely prevented CPE in 50% of the wells (NT_50_) were defined as the virus neutralization antibody.

### IFN-γ and IL-4 ELISpot assays

Lymphocytes isolated from the peripheral blood mononuclear cells (PBMCs) from inoculated monkeys at 2 weeks after the second immunization were plated at a density of 2×10^5^ cells per well in a 96-well ELISpot plate (Mabtech AB, Sweden), which had been pre-coated with IFN-γ or IL-4. Purified RBD antigen was added at a final concentration of 10 μg/ml to stimulate cytokine production, and the rest of the assay was carried out according to manufacturer instructions. Spot forming cells (SFCs) were counted using an automated ELISpot reader (AID ELISPOT reader-iSpot, AID GmbH, GER).

### Laboratory facility and ethics statement

The environment and housing facilities for rhesus macaques is in accordance with the National Standards of Laboratory Animal Requirements of China (GB 14925-2001). Animal studies were strictly conducted with the recommendations of the Veterinary Institute at the Academy of Military Medical Sciences and approved by the Animal Welfare and Ethics Committee (permit number SCXK-2012-017).

## References

[1] Zaki AM, van Boheemen S, Bestebroer TM, Osterhaus ADME, Fouchier RAM (2012). Isolation of a Novel Coronavirus from a Man with Pneumonia in Saudi Arabia. New England Journal of Medicine.

[2] Arabi YM, Arifi AA, Balkhy HH, Najm H, Aldawood AS, Ghabashi A, Hawa H, Alothman A, Khaldi A, Al Raiy B. (2014). Clinical course and outcomes of critically ill patients with Middle East respiratory syndrome coronavirus infection. Annals of internal medicine.

[3] Guery B, Poissy J, el Mansouf L, Séjourné C, Ettahar N, Lemaire X, Vuotto F, Goffard A, Behillil S, Enouf V, Caro V, Mailles A, Che D, Manuguerra J-C, Mathieu D, Fontanet A (2013). Clinical features and viral diagnosis of two cases of infection with Middle East Respiratory Syndrome coronavirus: a report of nosocomial transmission. The Lancet.

[4] ECDC. News and epidemiological updates.

[5] Cowling BJ, Park M, Fang VJ, Wu P, Leung GM, Wu JT (2015). Preliminary epidemiological assessment of MERS-CoV outbreak in South Korea, May to June 2015. Euro surveillance.

[6] Bolles M, Deming D, Long K, Agnihothram S, Whitmore A, Ferris M, Funkhouser W, Gralinski L, Totura A, Heise M, Baric RS (2011). A double-inactivated severe acute respiratory syndrome coronavirus vaccine provides incomplete protection in mice and induces increased eosinophilic proinflammatory pulmonary response upon challenge. Journal of virology.

[7] See RH, Petric M, Lawrence DJ, Mok CP, Rowe T, Zitzow LA, Karunakaran KP, Voss TG, Brunham RC, Gauldie J, Finlay BB, Roper RL (2008). Severe acute respiratory syndrome vaccine efficacy in ferrets: whole killed virus and adenovirus-vectored vaccines. The Journal of general virology.

[8] Spruth M, Kistner O, Savidis-Dacho H, Hitter E, Crowe B, Gerencer M, Bruhl P, Grillberger L, Reiter M, Tauer C, Mundt W, Barrett PN (2006). A double-inactivated whole virus candidate SARS coronavirus vaccine stimulates neutralising and protective antibody responses. Vaccine.

[9] Almazan F, DeDiego ML, Sola I, Zuniga S, Nieto-Torres JL, Marquez-Jurado S, Andres G, Enjuanes L (2013). Engineering a replication-competent, propagation-defective Middle East respiratory syndrome coronavirus as a vaccine candidate. mBio.

[10] Coleman CM, Liu YV, Mu H, Taylor JK, Massare M, Flyer DC, Glenn GM, Smith GE, Frieman MB (2014). Purified coronavirus spike protein nanoparticles induce coronavirus neutralizing antibodies in mice. Vaccine.

[11] Song F, Fux R, Provacia LB, Volz A, Eickmann M, Becker S, Osterhaus AD, Haagmans BL, Sutter G (2013). Middle East respiratory syndrome coronavirus spike protein delivered by modified vaccinia virus Ankara efficiently induces virus-neutralizing antibodies. Journal of virology.

[12] Wang L, Shi W, Joyce MG, Modjarrad K, Zhang Y, Leung K, Lees CR, Zhou T, Yassine HM, Kanekiyo M, Yang ZY, Chen X, Becker MM, Freeman M, Vogel L, Johnson JC (2015). Evaluation of candidate vaccine approaches for MERS-CoV. Nat Commun.

[13] Noad R, Roy P (2003). Virus-like particles as immunogens. Trends in Microbiology.

[14] Rodriguez-Limas WA, Sekar K, Tyo KE (2013). Virus-like particles: the future of microbial factories and cell-free systems as platforms for vaccine development. Curr Opin Biotechnol.

[15] Graham RL, Donaldson EF, Baric RS (2013). A decade after SARS: strategies for controlling emerging coronaviruses. Nat Rev Microbiol.

[16] Kushnir N, Streatfield SJ, Yusibov V (2012). Virus-like particles as a highly efficient vaccine platform: diversity of targets and production systems and advances in clinical development. Vaccine.

[17] Buckland B, Boulanger R, Fino M, Srivastava I, Holtz K, Khramtsov N, McPherson C, Meghrous J, Kubera P, Cox MM (2014). Technology transfer and scale-up of the Flublok recombinant hemagglutinin (HA) influenza vaccine manufacturing process. Vaccine.

[18] Shen HG, Halbur PG, Opriessnig T (2012). Prevalence and phylogenetic analysis of the current porcine circovirus 2 genotypes after implementation of widespread vaccination programmes in the USA. The Journal of general virology.

[19] Lopez-Macias C, Ferat-Osorio E, Tenorio-Calvo A, Isibasi A, Talavera J, Arteaga-Ruiz O, Arriaga-Pizano L, Hickman SP, Allende M, Lenhard K, Pincus S, Connolly K, Raghunandan R, Smith G, Glenn G (2011). Safety and immunogenicity of a virus-like particle pandemic influenza A (H1N1) 2009 vaccine in a blinded, randomized, placebo-controlled trial of adults in Mexico. Vaccine.

[20] Mortola E, Roy P (2004). Efficient assembly and release of SARS coronavirus-like particles by a heterologous expression system. FEBS Lett.

[21] Yao Y, Bao L, Deng W, Xu L, Li F, Lv Q, Yu P, Chen T, Xu Y, Zhu H, Yuan J, Gu S, Wei Q, Chen H, Yuen KY, Qin C (2014). An animal model of MERS produced by infection of rhesus macaques with MERS coronavirus. The Journal of infectious diseases.

[22] de Wit E, Rasmussen AL, Falzarano D, Bushmaker T, Feldmann F, Brining DL, Fischer ER, Martellaro C, Okumura A, Chang J, Scott D, Benecke AG, Katze MG, Feldmann H, Munster VJ Middle East respiratory syndrome coronavirus (MERS-CoV) causes transient lower respiratory tract infection in rhesus macaquesa. Proceedings of the National Academy of Sciences of the United States of America..

[23] Zhao J, Li K, Wohlford-Lenane C, Agnihothram SS, Fett C, Zhao J, Gale MJ, Baric RS, Enjuanes L, Gallagher T, McCray PB, Perlman S Rapid generation of a mouse model for Middle East respiratory syndrome. Proceedings of the National Academy of Sciences of the United States of America..

[24] Pascal KE, Coleman CM, Mujica AO, Kamat V, Badithe A, Fairhurst J, Hunt C, Strein J, Berrebi A, Sisk JM, Matthews KL, Babb R, Chen G, Lai KM, Huang TT, Olson W Pre- and postexposure efficacy of fully human antibodies against Spike protein in a novel humanized mouse model of MERS-CoV infection. Proceedings of the National Academy of Sciences of the United States of America..

[25] Papaneri AB, Johnson RF, Wada J, Bollinger L, Jahrling PB, Kuhn JH (2015). Middle East respiratory syndrome: obstacles and prospects for vaccine development. Expert Rev Vaccines.

[26] Muthumani K, Falzarano D, Reuschel EL, Tingey C, Flingai S, Villarreal DO, Wise M, Patel A, Izmirly A, Aljuaid A, Seliga AM, Soule G, Morrow M, Kraynyak KA, Khan AS, Scott DP (2015). A synthetic consensus anti-spike protein DNA vaccine induces protective immunity against Middle East respiratory syndrome coronavirus in nonhuman primates. Sci Transl Med.

[27] Slifka MK, Amanna I (2014). How advances in immunology provide insight into improving vaccine efficacy. Vaccine.

[28] Du L, Zhao G, Kou Z, Ma C, Sun S, Poon VK, Lu L, Wang L, Debnath AK, Zheng BJ, Zhou Y, Jiang S (2013). Identification of a receptor-binding domain in the S protein of the novel human coronavirus Middle East respiratory syndrome coronavirus as an essential target for vaccine development. Journal of virology.

[29] Mou H, Raj VS, van Kuppeveld FJ, Rottier PJ, Haagmans BL, Bosch BJ (2013). The receptor binding domain of the new Middle East respiratory syndrome coronavirus maps to a 231-residue region in the spike protein that efficiently elicits neutralizing antibodies. Journal of virology.

[30] Zhao G, Du L, Ma C, Li Y, Li L, Poon VK, Wang L, Yu F, Zheng BJ, Jiang S, Zhou Y (2013). A safe and convenient pseudovirus-based inhibition assay to detect neutralizing antibodies and screen for viral entry inhibitors against the novel human coronavirus MERS-CoV. Virology journal.

